# Whey protein hydrolysates enhance grapevine resilience to abiotic and biotic stresses

**DOI:** 10.3389/fpls.2025.1521275

**Published:** 2025-05-09

**Authors:** Esteban Alfonso, Wilfried Andlauer, Wolfram Manuel Brück, Markus Rienth

**Affiliations:** ^1^ HES-SO Changins, College of Viticulture and Enology, University of Applied Sciences and Arts Western Switzerland, Nyon, Switzerland; ^2^ HES-SO Valais-Wallis, Institute of Life Sciences, University of Applied Sciences and Arts Western Switzerland, Sion, Switzerland

**Keywords:** *Vitis vinifera*, biostimulant, protein hydrolysate, whey, abiotic stress, biotic stress

## Abstract

**Introduction:**

The growing need for sustainable viticulture has increased interest in biostimulants that enhance plant resilience to abiotic and biotic stresses. This study evaluates the efficacy of whey-derived protein hydrolysates (PHs) in improving *Vitis vinifera* cv. Cabernet Sauvignon tolerance to combined heat and drought stress and reducing pathogen infections.

**Methods:**

Potted grapevines were subjected to 40°C heat stress without irrigation and treated with either water or PHs. Physiological parameters as well as key stress- and photosynthesis-related genes expression were monitored. The antimicrobial effects of PHs against *Plasmopara viticola* and *Botrytis cinerea* were also assessed.

**Results:**

PHs-treated plants exhibited a faster recovery of photosynthetic activity than control plants and maintained normal sub-stomatal CO_2_ concentrations under combined abiotic stress. PHs treatment significantly upregulated heat stress-responsive genes (*HSFA2*, *HSP101*) and mitigated the stress-induced decline in photosynthesis-related genes (*LHCA3*, *RbcS*). Moreover, PHs significantly enhanced grapevine drought tolerance, as indicated by higher leaf water potential values and expression of drought-responsive genes (*NCED1*, *TIP2;1*). Additionally, PHs demonstrated a direct toxic effect on *P. viticola*, inhibiting zoospore germination and reducing sporulation on leaf discs, while reducing *B. cinerea* infection in berries when applied post-infection.

**Conclusion:**

In the tested conditions, whey PHs serve as effective biostimulants, enhancing grapevine resilience to combined drought and heat stress while providing protection against grapevine pathogens. Although further validation in vineyard conditions is needed, this dual benefit of PHs may propose a potential sustainable alternative to reduce chemical inputs in viticulture, contributing to more environmentally friendly agricultural practices.

## 1 Introduction

Grapevine (*Vitis vinifera* L.) is one of the most cultivated fruit crops worldwide, with a vineyard surface area of 7.2 million hectares in 2023 (OIV, 2024). Most of the winegrowing regions are situated between 30° and 50° latitude, both in the Northern and Southern hemisphere, where the average annual temperature ranges between 10°C and 20°C. The majority of these regions become increasingly impacted by climate change, leading to greater risks of extreme weather events, such as heat waves and droughts (Bernardo et al., 2018). These events are often interconnected and occur in combination, exposing plants to multiple stresses simultaneously. The impacts of these abiotic stresses can impair general grapevine physiology by reducing photosynthesis efficiency, leading to lower carbohydrate reserves, reduced fertility and flowering rates, and deteriorated berry quality (Greer and Weston, 2010; Rienth et al., 2021). Ultimately, this results in lower production yields and compromised wine quality. In the long term, many viticultural zones, particularly in Mediterranean climate regions, may face significant challenges in cultivating wine grapes unless growers and the scientific community collaborate on effective mitigation strategies (Hannah et al., 2013).

Moreover, the combination of elevated temperatures and erratic rainfall may lead to a general increase in the incidence and severity of disease outbreaks, creating additional challenges for vineyard pest management (Bois et al., 2017; Bove et al., 2020; Rienth et al., 2021). Most *V. vinifera* cultivars used for wine and table grapes production are highly susceptible to fungal and oomycete pathogens, such as *Botrytis cinerea* and *Plasmopara viticola*, which cause grey mold and downy mildew, respectively. Downy mildew is one of the most devastating grapevine diseases worldwide and its control requires high amounts of fungicide applications. In conventional viticulture, downy mildew is usually controlled by 7 to 15 applications of synthetic fungicides throughout the growing season (Gessler et al., 2011; Rienth et al., 2021). Organic grape production, where synthetic fungicides are not allowed, is still highly dependent on the application of copper-based formulations to control downy mildew. However, both strategies are considered as critical since they lead to the accumulation of harmful residues in soil and water, threatening biodiversity and delicate ecosystems (Droz et al., 2021; Karimi et al., 2021). Consequently, there is a growing imperative to explore sustainable alternatives that could minimize the reliance on synthetic chemicals while maintaining crop yield and quality.

Biostimulants are natural substances that positively influence plant physiology by enhancing nutrient uptake, promoting growth and improving resilience to biotic and abiotic factors without the environmental drawbacks associated with conventional chemical inputs (Van Oosten et al., 2017). Protein hydrolysates (PHs) are a mixture of peptides and amino acids derived from the enzymatic or chemical hydrolysis of proteins. These proteins are sourced from various materials, including animal by-products like whey or plant biomass (Pasković et al., 2024). This approach has gained popularity due to its economic, sustainable and eco-friendly method of recycling agricultural waste (Malécange et al., 2023). PHs have shown great efficiency as plant biostimulants, increasing the productivity and quality of a wide range of agronomic crops (Colla et al., 2017). Beyond their role in enhancing crop productivity, PHs offer numerous environmental benefits compared to conventional synthetic inputs. Their biodegradable nature prevents the accumulation of agrochemical residues in soil and water, reducing the risk of contamination compared to synthetic chemicals (Roche et al., 2024). Moreover, PHs have been shown to have a positive impact on soil health by enhancing microbial activity and increasing organic carbon mineralization, which could potentially benefit plant growth (Hellequin et al., 2018; Roche et al., 2024). Additionally, PHs can act as elicitors of plant defenses while also exhibiting direct antimicrobial activity, which could reduce reliance on synthetic fungicides and slow resistance development in plant pathogens caused by continuous fungicide use (Cappelletti et al., 2016; Colla et al., 2017). Finally, their application in viticulture aligns with sustainable agricultural practices by supporting a circular economy, transforming agricultural by-products into valuable biostimulants (Gedif and Tkaczewska, 2024). However, despite their promising advantages, further research is needed to evaluate their long-term environmental impacts and efficacy in large-scale vineyard applications.

Several studies have demonstrated that PHs can improve crop resilience to abiotic and biotic stresses. For instance, Meggio et al. (2020) showed that root application of collagen-derived PHs in *V. vinifera* cv. Sauvignon Blanc alleviated the consequences of water deficit by sustaining vegetative growth and limiting cell dehydration. Another study showed that casein- and soybean-derived PHs increased tolerance to water stress in *V. vinifera* cv. Corvina by reducing stomatal conductance and transpiration (Boselli et al., 2019). In addition to enhancing abiotic stress tolerance, PHs have also been shown to trigger defense responses in grapevine. Casein- and soybean-derived PHs, for instance, were shown to induce resistance against *P. viticola* by triggering a rapid increase in cytosolic Ca^2+^ followed by transcriptome reprogramming. This reprogramming led to the upregulation of several defense genes encoding pathogenesis-related (PR) proteins and stilbene synthase (STS) enzyme, involved in the biosynthesis of resveratrol, the main grapevine phytoalexin (Lachhab et al., 2014). Interestingly, the effect of casein- and soybean-derived PHs also extended to the control of *B. cinerea* on table and wine grapes (Lachhab et al., 2016).

Overall, studies on the effects of PHs on grapevine resilience to combined abiotic stresses are relatively scarce. This study aimed to characterize the biostimulant effect of PHs produced from whey, a by-product of the dairy industry (Andlauer et al., 2024), in improving grapevine resilience to combined heat and drought stress, as well as in promoting resistance to pathogens. For this purpose, experiments assessing grapevine physiological parameters and gene expression on plants subjected to combined heat and drought stress were conducted. The protective and curative effects of whey PHs against *P. viticola* and *B. cinerea* on both leaves and berries were also evaluated.

## 2 Materials and methods

### 2.1 Plant material and treatments

All experiments were conducted with cuttings of *Vitis vinifera* L. cv. Cabernet Sauvignon. The vines used in this work were propagated through hardwood cuttings. The wood was obtained from certified vineyards of the Agroscope clonal selection program. Cuttings were grown in pots of 1 L (Ø 13 cm) containing a peat-rich substrate mix (55% blonde peat, 10% compost, 10% coconut fiber, 15% topsoil and 10% perlite). After 14–16 weeks of vegetative growth under standard greenhouse conditions (20°C day/18°C night, 50-60% humidity with supplemental lighting from September to May: 100 W/m² for 12 h daily), the vines were transferred to climate chambers (Polyklima, Germany) under 25°C day/20°C night, 55% humidity and 14 h light/10 h darkness for 3–5 d to acclimate before the start of experiments.

For the abiotic stress experiments, plants of identical age (approximately 4 months old) and similar growth (20–22 leaves) were divided into two groups of 8 plants each. One group of plants was sprayed with whey PHs (at a concentration of 10%), and the other group was sprayed with water. In total, plants were subjected to three treatments over one week (on Monday, Wednesday and Friday). Three days later [the following Monday; 0 h post-stress (hps)], gas exchange parameters and chlorophyll fluorescence were measured for all plants (detailed parameters described in section 2.5). Subsequently, half of the PHs-treated and water-treated plants were then subjected to a combined heat and drought stress (40°C day/30°C night, no irrigation) for 5 d (Greer and Weston, 2010). These experimental conditions were designed to replicate the hot and dry climate typical of the southern Mediterranean region, characterized by prolonged periods of high temperatures and drought (Nielsen et al., 2024). These plants will be referred to as S-H_2_O and S-PHs. The other half remained at 25°C day/20°C night but were also not irrigated for 5 d (C-H_2_O and C-PHs plants). The last irrigation was performed at 0 hps, before starting the heat stress. Gas exchange parameters and chlorophyll fluorescence were measured at 24 hps, 96 hps and 120 hps of heat and drought stress. Then, S-H_2_O and S-PHs plants were allowed to recover at 25°C and all plants were irrigated at 100% field capacity. Photosynthetic parameters were then measured each day for 2 d during the recovery period [24 and 48 h recovery (hrec)]. The experiment was repeated three times independently under the same conditions.

For the *P. viticola* infection assays, leaf discs (Ø 18 mm) were punched out of leaves with a cork borer and placed on a wet filter paper in Petri dishes (Ø 90 mm). Petri dishes containing 10 leaf discs were then treated on their abaxial side with 5 mL of PHs at several concentrations using a Potter precision spray tower (Burkard Manufacturing Co Ltd, United Kingdom). Water and a 0.0625% copper hydroxide solution (Kocide^®^ Opti, Bayer, Switzerland) were used as negative and positive controls, respectively. Twenty-four hours later, leaf discs were inoculated with a *P. viticola* sporangia solution and sporulating area were quantified at 7 d post-inoculation (dpi). A total of 30 leaf discs per condition were used in each independent experiment. The assays were repeated three times independently under the same conditions.

### 2.2 Pathogens culture, inoculation and growth assessment

*Plasmopara viticola* (Berk. & M.A. Curtis; Berl & De Toni) sporangia were collected from sporulating lesions of artificially infected leaves of *V. vinifera* cv. Cabernet Sauvignon by vacuum aspiration using a filtered tip. A sporangia solution was generated and, using a Thoma cell (hemocytometer), adjusted to 2 x 10^5^ cells/mL in a 50 mL Falcon tube containing ultrapure water and gently stirred for 2 h at room temperature in darkness. As soon as the zoospores were released (5 x 10^4^ motile zoospores per mL), this suspension was used to inoculate leaf discs using a custom-made glass spray. Disease intensity was then assessed 7 d post-inoculation by measuring the leaf disc area covered by sporulation.

*Botrytis cinerea* strain BMM (Zimmerli et al., 2001) was grown on potato dextrose agar (PDA, 39 g L^-1^, Condalab, Spain) for 10 d in darkness at 22°C. Conidia were harvested in water and filtered through wool to remove hyphae.

### 2.3 Whey protein hydrolysates

The raw milk was purchased from a local cheese factory. Cheese production was carried out in the pilot plant of HES-SO Valais-Wallis, Institute of Life Technologies. Whey obtained after cheese production was centrifuged (CLARA 20 separation unit, Alfa Laval, Germany, with the following settings: 50°C, 11’130 g, 1.3 bar, flowrate 100 L h^-1^) and pasteurized (plate heat exchanger Rosista APV: 72°C, 30s). To increase protein and reduce mineral and lactose levels, the whey was ultrafiltered twice using a pilot unit (SW25 MMS, Tami membrane UF 3 kDa: 50 .C, TMP 1.5–2.5 bar, 400 L h^-1^, flux 8–9 L m^-2^ h). In the first step, 20 L of whey was concentrated to 1.5 L, then diluted with 20 L of demineralized water and reconcentrated to 2.7 L in the second step. The protein content of the whey concentrate was analyzed using the Kjeldahl method, following the ISO 8968-3:2007/IDF 20-3:2007 standard and applying a nitrogen-to-protein conversion factor of 6.38. The resulting concentrate was then hydrolyzed enzymatically for 4 h using a combination of the endopeptidase Alcalase^®^ Pure 2.4 L and the exopeptidase Flavourzyme^®^ 1000 L (Novozymes AG, Denmark). Whey concentrates (900 mL, equivalent to 8.3 g of protein) were incubated with 225 mg of Flavourzyme (225 LAPU) = 180 μL of Flavourzyme enzyme preparation and 225 mg of Alcalase (21.6 Anson units/g = 4.86 Anson units = 2673 IU) = 180 μL of Alcalase enzyme preparation. A carbonate/bicarbonate buffer (90 mL, 0.1 mol L^-1^, pH 7.5) was added, and the temperature was maintained at 50°C. The pH was kept at 7.5 using NaOH (1 mol L^-1^) with an automated pump. The resulting whey protein hydrolysates were then stored at 4°C until usage.

For foliar application, whey hydrolysates were diluted with Milli-Q water to a final concentration of 10%. Preliminary trials indicated that application of lower concentrations resulted in insufficient physiological responses (data not shown). This concentration was thus chosen for all the experiments presented in this study.

### 2.4 Toxicity assays on *P. viticola* and *B. cinerea*

The direct effect of PHs on *P. viticola* was analyzed in two different ways. Firstly, the inhibition of zoospore motility was evaluated by mixing the same volume of PHs and sporangia suspension, for 2 h at room temperature. Both the PHs solution and sporangia suspension were prepared (double concentrated) to obtain a final mixture with the desired concentrations. The final concentration of sporangia was 2 x 10^4^ cells/mL and the final concentration of PHs was 10%. Water and Kocide^®^ Opti (K.O., concentrated at 0.0625%) were used as negative and positive controls, respectively. The number of motile zoospores per min was calculated using a Thoma cell. For each independent experiment, three independent mixtures were prepared per condition, and three observations were made per mixture (n=9). The same mixtures were then used to infect leaf discs to evaluate whether the sporangia were able to develop an infection. A total of 20 leaf discs per condition and per independent experiment were inoculated by depositing four 20 µL drops on the abaxial side of leaf discs placed in Petri dishes. At seven days post-inoculation, leaf discs were collected and placed in a 15 mL Falcon tube filled with 2 mL water and vortexed. Sporangia released in water were then counted using a Thoma cell and results reported as number of sporangia per mL. The experiments were repeated three times independently under the same conditions.

For *in vitro* antifungal assays, plugs (Ø 5 mm) were taken from a 10-d-old *B. cinerea* culture on PDA and transferred to Petri dishes (Ø 60 mm) supplemented with 10% PHs in PDA. Control plates were supplemented with water. Radial growth was assessed on 10 plates per condition and per experiment after 48 h of incubation at 23°C in darkness. The assays were repeated three times independently under the same conditions. Mycelial growth inhibition was calculated using the following formula: MGI % = ((C–T)/C) x 100, where C is the average colony diameter on control plates, and T is the average colony diameter on treated plates.

For infection assays on fruits, berries from *V. vinifera* cv. Chasselas were collected at the institute experimental vineyard located in Nyon, Switzerland. Berries with 2 mm pedicel were submerged in 1% sodium hypochlorite for 3 min to surface sterilize them, then rinsed three times with distilled water and air-dried under a laminar flow hood. After being separated into four groups of 30 berries each and arranged in Petri dishes (Ø 90 mm), berries were wounded with one wound site at their equatorial line using a sterile blade. Twenty µL of 10% PHs was pipetted onto the wound site. Treatments were applied 24 h prior or after *B. cinerea* infection to evaluate the protective and curative effects of PHs, respectively. Control groups were treated with water. Ten µL of *B. cinerea* infection solution (10^4^ conidia/mL) was pipetted onto the wound sites. Berries in Petri dishes were placed in a humidified plastic box and kept at 23°C for 4 d. Disease symptoms were photographed, and the lesion areas were quantified using ImageJ software. The experiment was repeated three times independently under the same conditions.

### 2.5 Plant physiological parameters measurements

Net photosynthesis rate (*A*), stomatal conductance (*gs*) and sub-stomatal CO_2_ concentration (*Ci*), were measured using a portable photosynthesis system (CIRAS-3, PP Systems, USA) with the following settings: photosynthetically active radiation (PAR) at 1800 mmol m^-2^ s^-1^, temperature at 25°C for unstressed leaves or 40°C for heat-stressed leaves and CO_2_ reference at 400 ppm. Chlorophyll fluorescence was measured using the CFM-3 chlorophyll fluorescence module to assess the maximum quantum efficiency of photosystem II (Fv/Fm ratio). For that, leaves were dark-adapted for 20 min before measurements. All measurements were conducted at 14:00 h on the fifth leaf from the apex of each plant. Four measurements per experiment were made with leaves from different plants. The experiment was repeated three times independently under the same conditions.

Predawn leaf water potential (Ψpd) was measured on fully developed leaves (one per plant) using a Scholander pressure chamber (PMS Instrument, USA). Measurements were conducted at 0 hps (before starting the heat stress), 72 hps, 120 hps and 48 hrec. All measurements were conducted at 09:00 h, just before the lights in the climate chamber turned on. Four measurements per experiment were made with leaves from different plants. The experiment was repeated three times independently under the same conditions.

### 2.6 RNA extraction, cDNA synthesis and RT-qPCR

For the abiotic stresses experiment, one leaf per plant (four in total per independent experiment) were harvested at each sampling day, pooled and immediately frozen in liquid nitrogen. Leaves were then ground in liquid nitrogen using a pestle and mortar. For *P. viticola* infection assays, 12 leaf discs (Ø 18 mm) from four plants were harvested for each condition and per independent experiment. Leaf discs were pooled, transferred to 2 mL microtubes, immediately frozen in liquid nitrogen, and subsequently ground using a TissueLyser II (Qiagen). The experiments were repeated three times independently under the same conditions.

For all samples, total RNA was extracted from 100 mg of powdered tissue using the Spectrum™ Plant Total RNA Kit (Merck), following manufacturer’s instructions. For cDNA synthesis, 500 ng of total RNA was reverse-transcribed using M-MLV reverse transcriptase (Invitrogen) in a final volume of 15.25 µL. Each cDNA sample was generated in triplicate from a single RNA pool and diluted eightfold with water. Quantitative reverse transcription PCR analysis was performed in a final volume of 10 µL containing 1 µL of cDNA, 0.2 µM of each primer, nuclease-free water and 5 µL of GoTaq^®^ qPCR Master Mix (Promega). Reactions were performed using an CFX96™ Real-Time System PCR machine (Bio-Rad) with the following program: 95°C for 2 min, then 40 cycles of 20 s at 95°C and 20 s at 60°C.

Relative gene expression of the target genes were calculated by using the 2^-ΔΔCt^ method (Livak and Schmittgen, 2001) and presented as fold change relative to the control. Ct values were normalized to the expression of the reference gene *EF1a*. The ΔΔCt values were obtained by normalizing ΔCt values to the mean ΔCt of control treatment (C-H_2_O at 0 hps for abiotic stress experiments and H_2_O/H_2_O for biotic stress experiments). Primers used in this study are listed in **Supplementary Table 1**.

### 2.7 Statistical analyses

Data were analyzed using GraphPad Prism software v.9.0. Normal distribution and variance homogeneity of data were first evaluated using R software v.3.6.0 with the Shapiro-Wilk test and Levene’s test, respectively. If not normal, data were log-transformed to enable analyses with parametric tests.

For physiological parameters and gene expression analyses, data were analyzed using one-way ANOVA to assess differences among treatments within the same timepoint. When significant differences were detected (P < 0.05), Tukey’s Honest Significant Difference *post-hoc* test was applied to determine pairwise comparisons between treatments. Data are presented as means ± SEM of three independent experiments, and statistical significance is indicated by different letters.

## 3 Results

### 3.1 Whey protein hydrolysates alleviate decreases in photosynthesis under combined heat and drought stress

**Figure 1:**
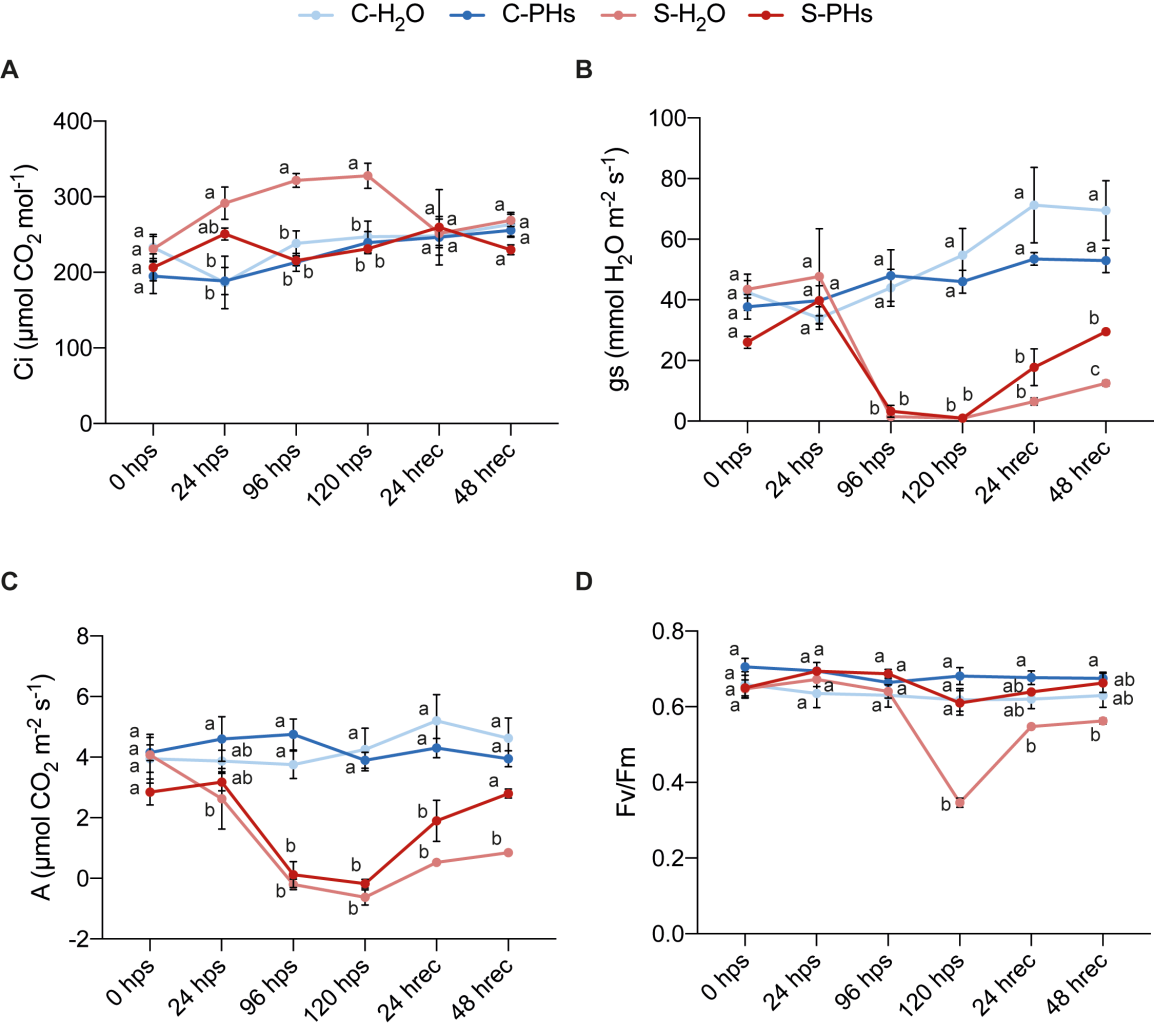
Physiological parameters measured before, during and after combined stress. *V. vinifera* plants were treated either with water or PHs and subjected to a heat stress of 40°C without irrigation (S-H_2_O/PHs) or remained at 25°C (C-H_2_O/PHs). Sub-stomatal CO_2_ concentration (*Ci*) **(A)**, stomatal conductance (*gs*) **(B)**, net photosynthesis rate (*A*) **(C)** and chlorophyll fluorescence (Fv/Fm) **(D)** were measured before, during and after stress. Data represent means ± SEM of three independent experiments (n=4 per experiment). Different letters indicate significant differences at *P* < 0.05 (One-Way ANOVA followed by Tukey’s Honest Significant Difference test) between treatments within the same timepoint. Abbreviations: hps, h post-stress; hrec, h of recovery.

We monitored key physiological parameters before, during, and after stress application to evaluate the effects of PHs treatment. Under normal growth conditions, PHs treatment did not induce significant changes in sub-stomatal CO_2_ concentration (*Ci*), stomatal conductance (*gs*), net photosynthesis rate (*A*), or PSII efficiency (Fv/Fm) of C-PHs plants compared to C-H_2_O plants (**Figure 1**). However, under the same conditions, C-PHs plants exhibited a higher growth rate compared to C-H_2_O plants and stressed plants (**Supplementary Figure 1**), demonstrating the biostimulant effect of whey PHs. During heat stress, *Ci* rose sharply in S-H_2_O plants and remained elevated until 120 hps, unlike in S-PHs plants and control plants (**Figure 1A**). *Ci* levels of S-H_2_O plants returned to normal after 24 h of recovery (24 hrec) and remained stable 48 h post-recovery. Meanwhile, both *gs* and *A* dropped sharply after 24 h of stress and remained close to zero until the end of the stress period in S-H_2_O and S-PHs plants. PHs-treated plants recovered more effectively after stress cessation compared to S-H_2_O plants (**Figures 1B, C**). PSII efficiency, indicated by the Fv/Fm ratio, also decreased significantly in S-H_2_O plants at 120 hps, reaching 0.37, while S-PHs plants maintained levels comparable to controls that remained at 25°C. At 24 hrec and 48 hrec, S-H_2_O plants showed partial recovery, although their Fv/Fm ratio remained slightly lower than those of other treatment groups (**Figure 1D**).

**Figure 2:**
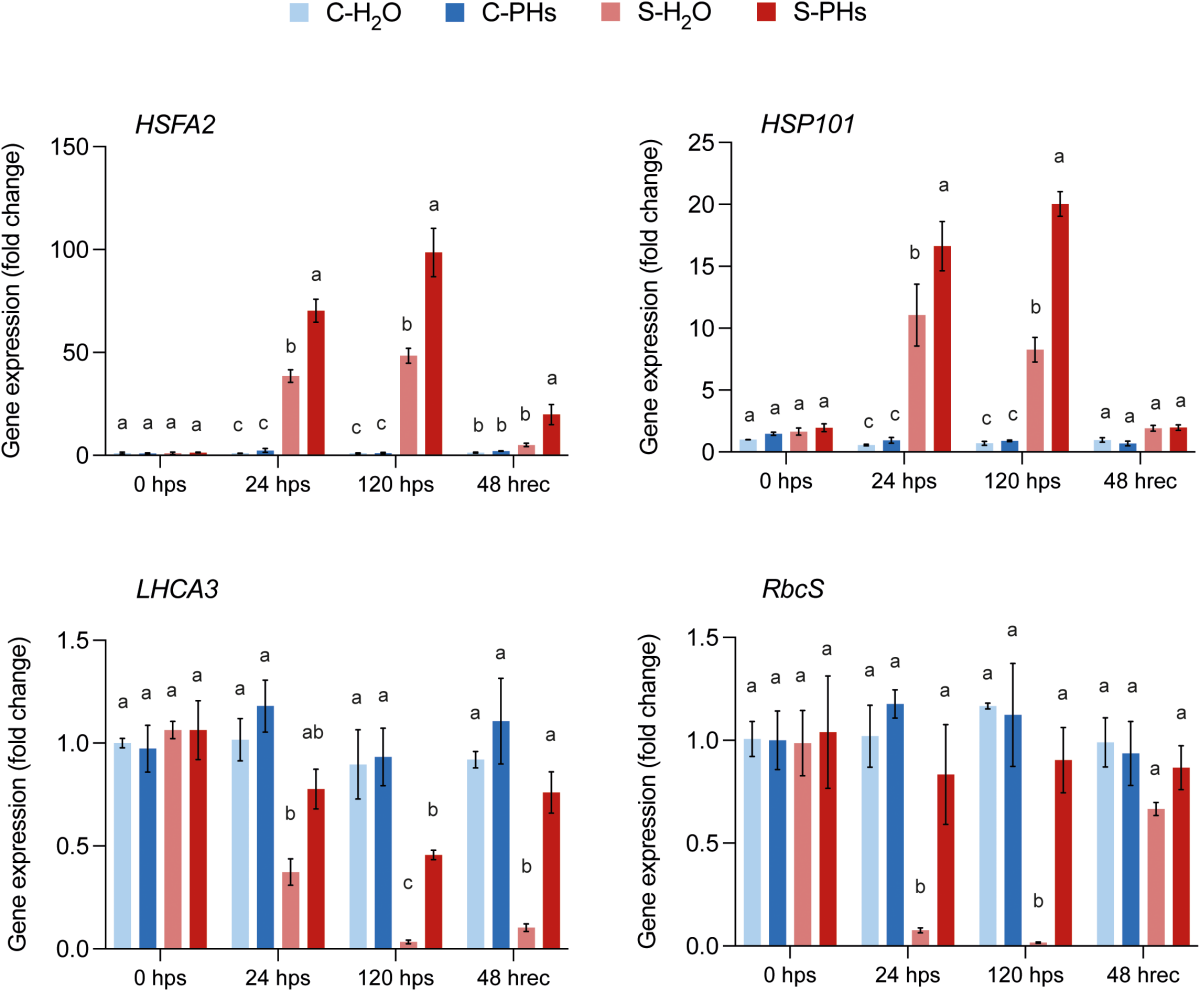
Expression analysis of heat stress-responsive and photosynthesis-related genes. Data represent means ± SEM of three independent experiments (n=3), each consisting of a pool of four leaves. Different letters indicate significant differences at *P* < 0.05 (One-Way ANOVA followed by Tukey’s Honest Significant Difference test) between treatments within the same timepoint.

To better understand and confirm the physiological responses, we monitored the expression of genes involved in the heat stress response in grapevine leaves, including the transcription factor *HSFA2* and *HSP101*, which encodes a heat-shock protein (Pillet et al., 2012; Ju et al., 2021). We also measured the expression of two photosynthesis-related genes: *LHCA3*, which codes for a chlorophyll-binding protein in the light-harvesting complex of PSI, and *RbcS*, encoding the small subunit of Rubisco. Both *HSFA2* and *HSP101* were upregulated in response to combined stress at 24 hps and 120 hps (**Figure 2**; **Supplementary Figure 2**). Notably, *HSFA2* and *HSP101* expression levels were significantly higher in S-PHs plants than in S-H_2_O plants at both timepoints, with *HSFA2* expression remaining significantly more elevated after 48 h of recovery at 25°C (**Figure 2**). In contrast, *LHCA3* and *RbcS* transcript levels were constitutively expressed before heat stress (0 hps) across all treatments. However, their expression drastically declined after 24 h of stress exposure in S-H_2_O plants (**Figure 2**), indicating stress-induced downregulation. The expression level of *LHCA3* was significantly less reduced in S-PHs plants, while *RbcS* expression was not impacted (**Figure 2**). Notably, the expression of these genes was not impacted across all conditions after 24 h of PHs treatment alone (**Supplementary Figure 3**).

### 3.2 Whey protein hydrolysates mitigate the adverse effects of drought stress

**Figure 3:**
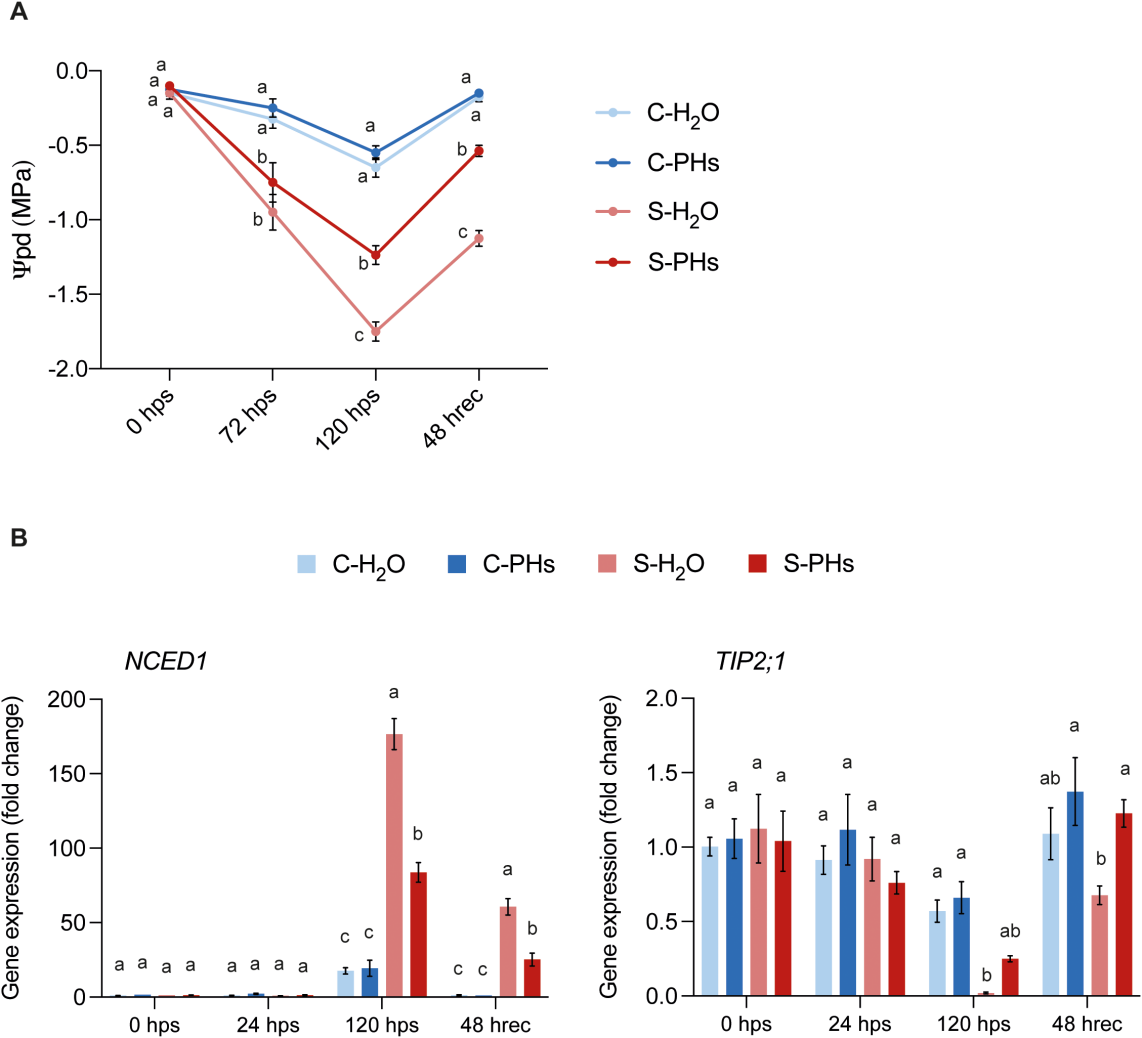
Water potential measurements and drought-responsive gene expression. **(A)** Predawn leaf water potential measurements. Data represent means ± SEM of three independent experiments (n=4 per experiment). Different letters indicate significant differences at *P* < 0.05 (One-Way ANOVA followed by Tukey’s Honest Significant Difference test) between treatments within the same timepoint. **(B)** Gene expression analysis of drought-responsive genes. Data represent means ± SEM of three independent experiments (n=3), each consisting of a pool of four leaves. Different letters indicate significant differences at *P* < 0.05 (One-Way ANOVA followed by Tukey’s Honest Significant Difference test) between treatments within the same timepoint.

To characterize the effects of PHs treatment on drought-induced responses in grapevine, we measured leaf water potential before, during, and after stress application. To assess plant water stress status, predawn leaf water potential (Ψpd) was measured. At 120 hps, S-H_2_O plants exhibited severe drought stress, with an average Ψpd of -1.75 MPa (**Figure 3A**). In contrast, S-PHs plants, although still experiencing severe drought stress, showed a significantly higher water status, with an average Ψpd of -1.23 MPa. Control plants maintained at 25°C without irrigation experienced moderate drought stress, with Ψpd values of -0.65 MPa for C-H_2_O plants and -0.55 MPa for C-PHs plants. After 48 h of recovery, Ψpd in S-PHs plants rose to -0.53 MPa, while S-H_2_O plants remained significantly more stressed with a Ψpd of -1.125 MPa (**Figure 3A**).

To assess the molecular response to drought stress, we monitored the expression of two key drought-responsive genes, *NCED1* and *TIP2;1*. *NCED1* expression was significantly higher in S-H_2_O plants than in S-PHs plants at 120 hps and after 48 h recovery (**Figure 3B**). Both C-H_2_O and C-PHs plants exhibited a similar increase in *NCED1* expression at 120 hps compared to earlier timepoints. However, this increase was not significantly influenced by PHs treatment (**Figure 3B**). In contrast, *TIP2;1* expression was significantly downregulated in S-H_2_O plants at 120 hps and 48 hrec in comparison to control plants (**Figure 3B**).

### 3.3 Treatment with whey protein hydrolysates reduces *Plasmopara viticola* infection

**Figure 4:**
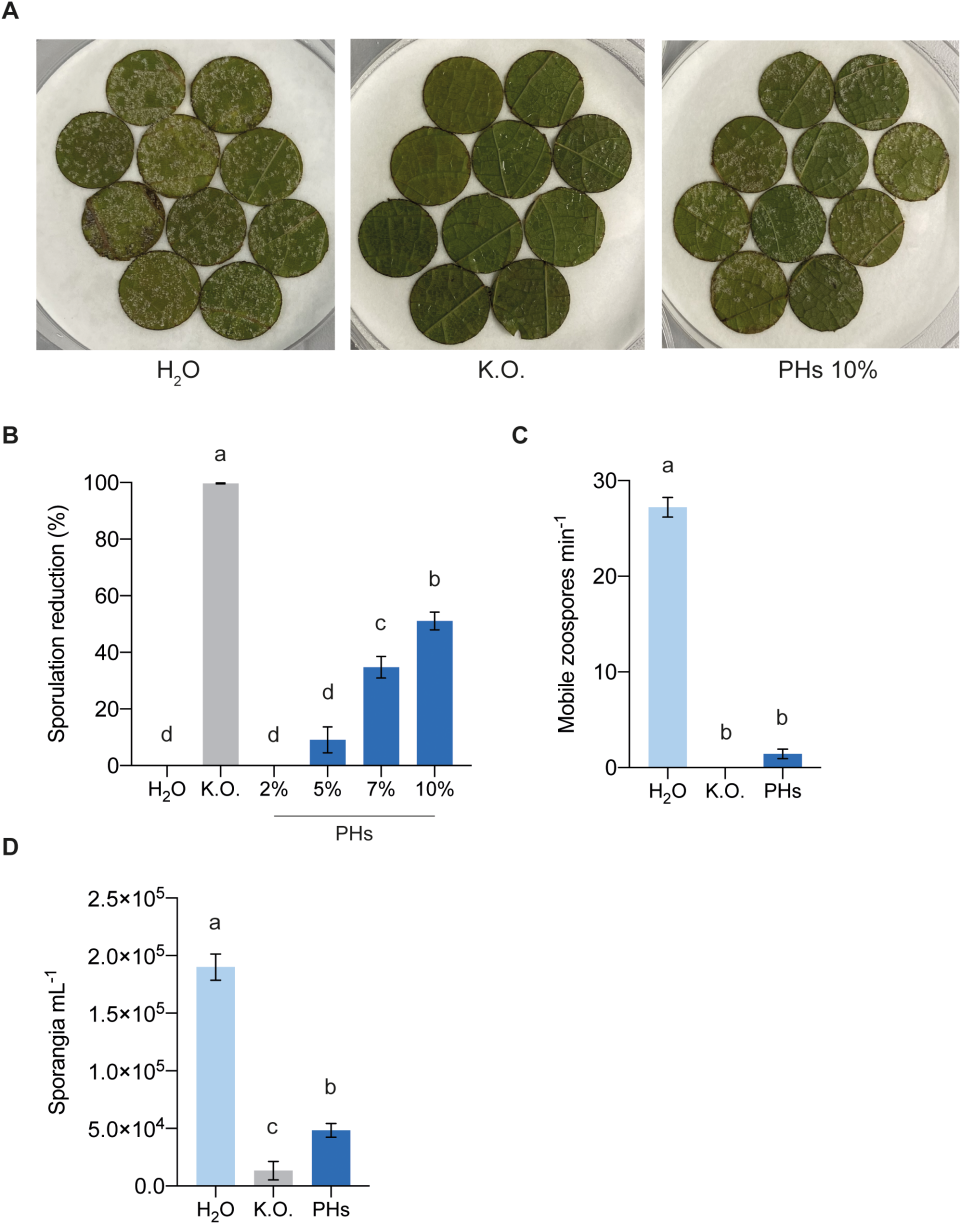
Effects of whey PHs on *P. viticola*. **(A)** Photographs of sporulating leaf discs treated with water (H_2_O), Kocide Opti (K.O.) and whey protein hydrolysates (PHs) at 7 dpi. **(B)** Effect of 24 h PHs treatment on *P. viticola* infection. Sporulating areas were quantified 7 dpi. Data represent means ± SEM of three independent experiments (n=30 per experiment). Different letters indicate significant differences at *P* < 0.05 (One-Way ANOVA followed by Tukey’s Honest Significant Difference test). **(C)** Motile zoospores per min counted after incubation of sporangia for 2 h with the different treatments. Data represent means ± SEM of three independent experiments (n=9 per experiment). Different letters indicate significant differences at *P* < 0.05 (One-Way ANOVA followed by Tukey’s Honest Significant Difference test). **(D)** Number of sporangia per mL retrieved on sporulating leaf discs. Data represent means ± SEM of three independent experiments (n=20 per experiment). Different letters indicate significant differences at *P* < 0.05 (One-Way ANOVA followed by Tukey’s Honest Significant Difference test).

To evaluate the potential of whey PHs in eliciting resistance against *P. viticola* infection in *V. vinifera* cv. Cabernet Sauvignon, leaf discs were treated with water, Kocide^®^ Opti (K.O., a copper hydroxide solution) and increasing concentrations of PHs. Twenty-four hours later, leaf discs were inoculated with *P. viticola* and sporulating areas were quantified at 7 dpi to determine the percentage reduction in sporulation (**Figure 4A**). As expected, treatment with K.O. completely inhibited sporulation development. Among the different concentrations tested, the 10% PHs treatment showed the greatest efficacy, reducing sporulation by 51% compared to the water-treated control. Treatments with 2%, 5% and 7% PHs resulted in sporulation reductions of 0%, 10% and 35%, respectively (**Figure 4B**). These results demonstrate the potential of whey PHs to reduce *P. viticola* infection in a dose-dependent manner, with 10% PHs exhibiting the highest efficacy.

To determine whether whey PHs exert a direct toxicity on *P. viticola*, we assessed their impact on sporangial germination by analyzing the number of motile zoospores after treatment. Incubation of sporangia with PHs significantly inhibited the release of motile zoospores, similarly to the positive control treated with K.O. (**Figure 4C**). Next, to determine whether sporangia and zoospores exposed to PHs retained their infectivity, the incubated sporangia were used to inoculate fresh leaf discs. Seven days post-inoculation, sporulation was quantified by collecting and counting sporangia. Leaf discs inoculated with PHs-treated sporangia exhibited significantly reduced sporulation compared to the water control, with slightly higher levels than the K.O. control (**Figure 4D**).

**Figure 5:**
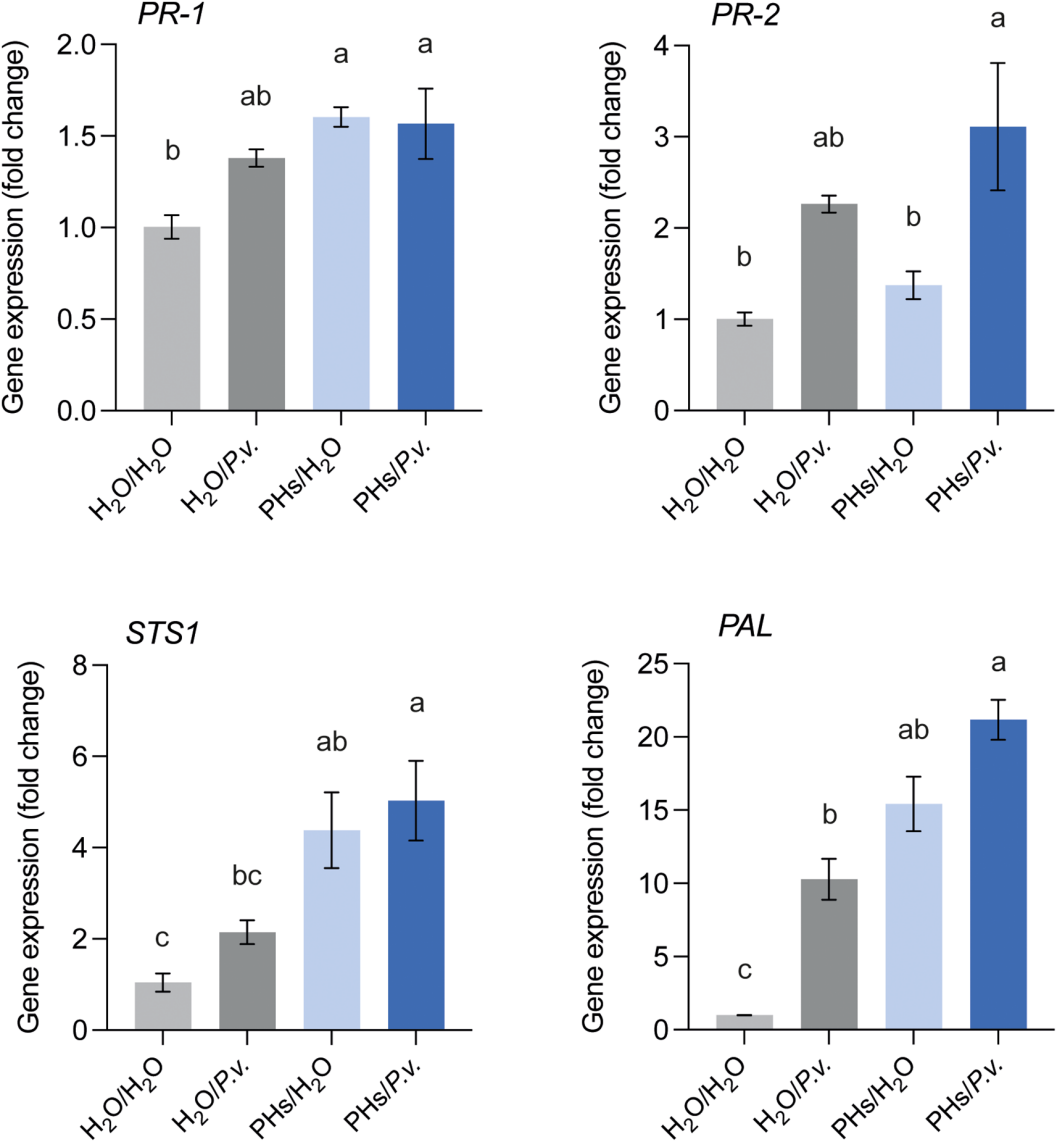
Defense gene expression analysis in response to whey PHs and *P. viticola* infection. Leaf discs were either treated with water (H_2_O) or whey PHs for 24 h and inoculated with water or *P. viticola* (*P.v.*) for an additional 24 h. Data represent means ± SEM of three independent experiments (n=3), each consisting of a pool of 12 leaf discs. Different letters indicate significant differences at *P* < 0.05 (One-Way ANOVA followed by Tukey’s Honest Significant Difference test).

In addition, we explored their potential role as elicitors of grapevine immune responses. To assess this, leaf discs were either treated with water or PHs for 24 hours, followed by inoculation with water or *P. viticola* sporangia for an additional 24 hours. We monitored the expression of two pathogenesis-related genes, the salicylic acid-responsive gene *PR-1* and the β-1,3-glucanase *PR-2*, as well as two genes involved in the phenylpropanoid pathway (*STS1* and *PAL*). Leaf discs treated with PHs and inoculated with water (PHs/H_2_O) exhibited a higher expression of *PR-1*, *STS1* and *PAL* in comparison to the water-inoculated control (H_2_O/H_2_O) (**Figure 5**). In contrast, *PR-2* was rather upregulated in response to *P. viticola* infection and not by the PHs treatment. Prior treatment with PHs followed by infection with *P. viticola* (PHs/*P.v.*) did not enhance the expression of any of the four genes compared to the other conditions.

### 3.4 Whey protein hydrolysates impact *Botrytis cinerea* infection on grape berries

**Figure 6:**
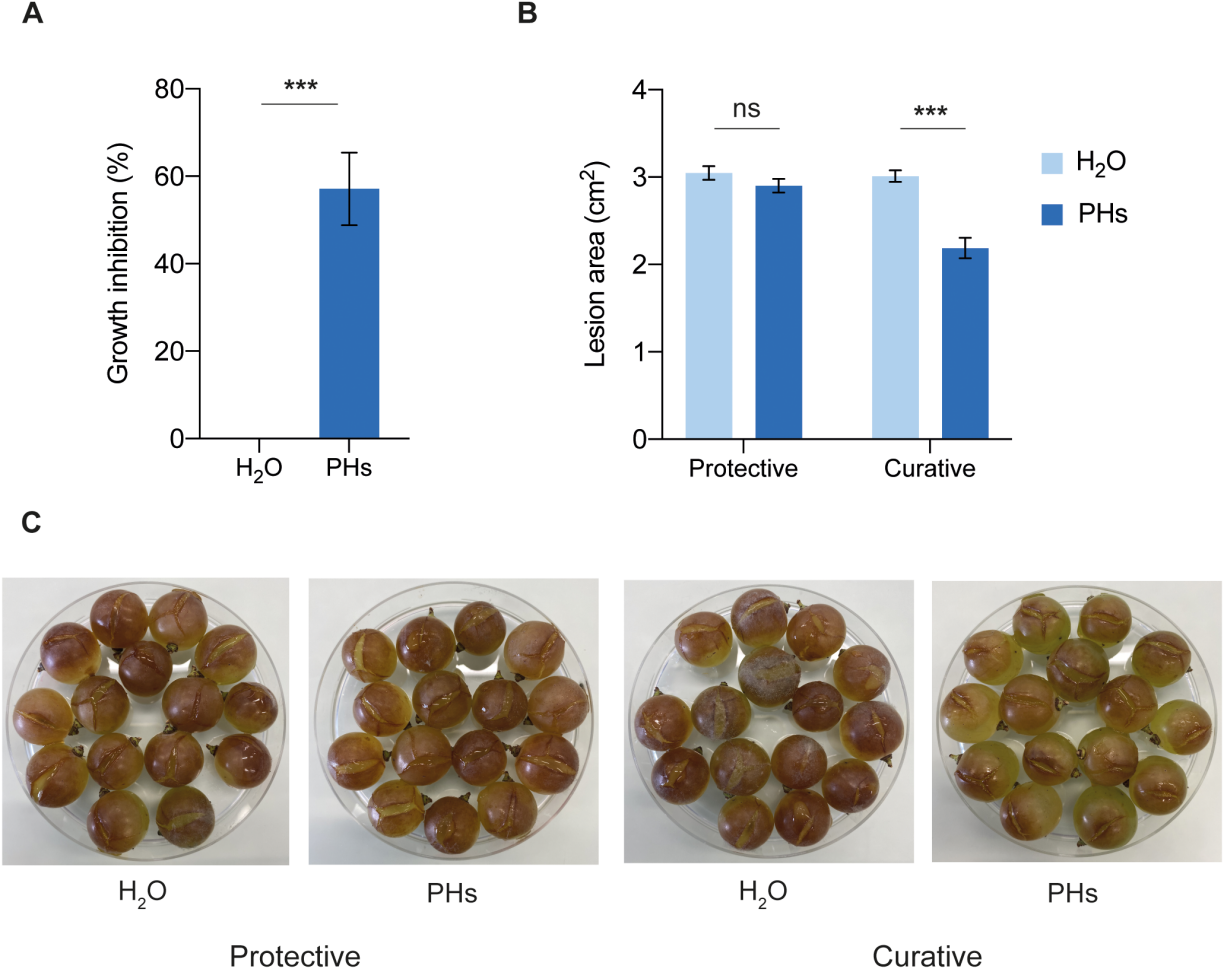
Effects of whey PHs on *B. cinerea*. **(A)** *In vitro* growth inhibition assay. PDA medium was supplemented with water or 10% PHs and *B. cinerea* radial growth was measured after 48 h of incubation. Data represent means ± SEM of three independent experiments (n=10 per experiment). Significant difference between treatments is indicated (Welch’s two samples t-test, ***P < 0.001). **(B)** Berries were treated with water or 10% PHs 24 h before (protective) or 24 h after (curative) *B. cinerea* infection. Lesion areas on berries were measured after 4 (d) Data represent means ± SEM of three independent experiments (n=30 per experiment). Significant differences are indicated (Welch’s two samples t-test, ***P < 0.001, ns: not significant). **(C)** Photographs of infected berries at 4 dpi.

To determine the antifungal activity of whey PHs against *B. cinerea*, we conducted *in vitro* growth assays on agar plates supplemented with PHs. On these plates, *B. cinerea* growth was inhibited by 57% compared to the water-treated control (**Figure 6A**). To evaluate the *in vivo* antifungal effect of PHs on *B. cinerea*, *V. vinifera* cv. Chasselas grape berries were treated with PHs either 24 h before or 24 h after infection. This approach aimed to assess the protective and curative potential of PHs in reducing *B. cinerea* symptoms on grape berries. Treatment with PHs applied 24 h before infection did not reduce *B. cinerea* growth on berries. However, when applied 24 h after infection, PHs significantly reduced *B. cinerea* symptoms (**Figures 6B, C**).

## 4 Discussion

### 4.1 Whey PHs application enhances grapevine resilience to combined heat and drought stress

This study investigates the potential of whey PHs as a biostimulant to alleviate abiotic stress in *V. vinifera* cv. Cabernet Sauvignon under controlled conditions. While many previous studies have explored the effects of biostimulants on plant growth, nutrient uptake and resistance to single stress (Jindo et al., 2022), few have specifically examined the role of whey PHs in grapevine under combined heat and drought stress conditions. As plants are often exposed to multiple environmental stresses simultaneously, understanding their responses to both heat and drought stress is crucial for developing efficient adaptation strategies (Zandalinas et al., 2022).

#### 4.1.1 Whey PHs mitigate stress-induced photosynthetic limitations

The overall trends in physiological responses (summarized in **Supplementary Table 2**) demonstrate that PHs treatment mitigated stress effects and accelerated recovery. Our results indicate that the inhibition of photosynthesis in stressed grapevines was primarily driven by non-stomatal limitations, as evidenced by the sharp rise in *Ci* observed in S-H_2_O plants, which remained elevated until the end of the stress period (**Figure 1**). This suggests that the decline in net photosynthesis (*A*) was not due to CO_2_ limitation but rather to biochemical constraints, likely linked to Rubisco deactivation. This is consistent with previous studies showing that heat stress above 40°C inhibits Rubisco activity and disrupts carbon assimilation in grapevines (Wang et al., 2010; Luo et al., 2011). In contrast, PHs-treated plants maintained *Ci* levels similar to those of control plants, despite experiencing a comparable decrease in *gs* and *A* following stress application. This suggests that PHs played a role in preserving CO_2_ fixation capacity and mitigating metabolic limitations, possibly through the stabilization of Rubisco function. In support of this, we showed that PHs treatment mitigated the decrease of *LHCA3* and *RbcS* expression observed in S-H_2_O plants during stress (**Figure 2**). These genes are essential for photosynthesis and decreases in their expression are associated with photosynthesis disruption (Petit et al., 2009). The sustained expression of *RbcS* in PHs-treated plants suggests that PHs help maintain Rubisco activity, preventing its deactivation and ensuring a more stable CO_2_ fixation capacity under stress compared to S-H_2_O plants.

Our results also suggest that PHs treatment contributed to the protection of PSII integrity. Indeed, the Fv/Fm ratio measured in S-PHs plants indicates that no damage to PSII reaction centers occurred, potentially facilitating a more efficient post-stress recovery. In contrast, the Fv/Fm ratio in S-H_2_O plants dropped to 0.37 at 120 hps (**Figure 1D**), indicating severe stress-induced photoinhibition. One potential explanation is that PHs mitigated oxidative damage to PSII reaction centers, as excessive reactive oxygen species (ROS) accumulation is a cause of heat-induced photoinhibition (Allakhverdiev et al., 2008; Zhang and Sharkey, 2009). These findings align with previous studies demonstrating that application of PHs improved PSII efficiency under similar abiotic stress conditions. Recently, Francesca et al. (2022) reported that PHs mitigated the negative effects of heat, drought and combined stress by increasing photosynthesis efficiency and modulating ROS, proline and soluble sugars in tomato plants. Another study showed that application of PHs from vegetal origin improved PSII efficiency and induced a higher accumulation of proline under combined heat and drought stress in soybean, chickpea and chilli plants, leading to increased yields (Mamatha et al., 2023).

In addition, the higher upregulation of *HSFA2* and *HSP101* in S-PHs plants may also have contributed to the stabilization of PSII-associated proteins and the overall maintenance of photosynthetic function under stress. Indeed, higher accumulation of heat shock proteins in plants helps preserve cellular functions and protect the photosynthesis apparatus from heat-induced damages (Wang et al., 2004). Our results suggest that PHs primed the heat stress response, thereby promoting cellular protection mechanisms that enhanced stress tolerance. A recent study reported higher expression of several *HSPs* in maize plants subjected to heat stress and pretreated with a commercial PHs-based biostimulant, supporting our findings (Vaseva et al., 2022).

These findings highlight the potential of whey PHs as a biostimulant that reduces heat-induced metabolic constraints, protects the photosynthetic machinery, and promotes faster post-stress recovery in plants facing severe abiotic stress. Further studies are needed to elucidate the precise molecular and biochemical mechanisms underlying these effects.

#### 4.1.2 Whey PHs enhance drought resilience and accelerate recovery

PHs-treated plants exhibited a significantly higher predawn leaf water potential during stress compared to non-treated plants (**Figure 3A**). As predawn leaf water potential serves as a reliable indicator of plant water status under drought conditions, our data suggest that PHs improved water retention and/or uptake. Recovery following stress cessation is crucial for plant survival and subsequent productivity. Our findings indicate that S-PHs plants regained water potential more effectively than S-H_2_O plants. This suggests that PHs not only alleviated drought stress during its occurrence but also accelerated recovery, possibly by enhancing water uptake mechanisms and reducing membrane damage caused by dehydration. In line with this, a recent study reported that PHs application alleviated the effects of water deficit in *V. vinifera* cv. Sauvignon Blanc by reducing the extent of cell dehydration (Meggio et al., 2020).

Interestingly, despite the higher water status observed in PHs-treated plants, *gs* was similarly reduced in both S-PHs and S-H_2_O plants during stress (**Figure 1B**), suggesting that PHs did not prevent stomatal closure under severe water deficit. However, upon rewatering, PHs-treated plants recovered their *gs* more effectively than non-treated plants, indicating a faster restoration of stomatal function. The accelerated recovery of *gs* could be explained by the lower expression of *NCED1* in S-PHs plants compared to S-H_2_O plants at 120 hps and 48 h after recovery (**Figure 3B**). *NCED1* encodes a 9-cis-epoxycarotenoid dioxygenase enzyme, which is involved in the biosynthesis of abscisic acid (ABA), an important regulator of stomatal closure upon drought stress in *V. vinifera* (Lehr et al., 2022). Consequently, its reduced expression suggests a more rapid decline in ABA levels after stress cessation, which would allow stomata to reopen more efficiently and thus *gs* to increase. It would be interesting to quantify ABA levels in PHs-treated plants facing drought conditions to gain a better understanding of this response. Notably, the high upregulation of *NCED1* in S-H_2_O plants at 120 hps is consistent with previous studies showing that this gene is highly activated in response to drought stress (Soar et al., 2004; Rossdeutsch et al., 2016; Lehr et al., 2022). Since PHs-treated plants exhibited lower *NCED1* expression than S-H_2_O plants, this suggests that PHs may contribute to improved water status by reducing the need for excessive ABA signaling. This indicates that PHs-treated plants experienced less severe drought stress or employed alternative protective strategies such as enhanced osmolyte accumulation or improved root water uptake. In line with this hypothesis, several studies reported increased accumulation of osmoprotectants after PHs application in various crops (Agliassa et al., 2021; Liatile et al., 2022).

The gene *TIP2;1*, encoding an aquaporin involved in intracellular water transport, has been shown to be a significant marker for drought-induced changes in *V. vinifera* (Pou et al., 2013). In contrast with *NCED1*, *TIP2;1* expression was significantly downregulated in S-H_2_O plants but maintained at higher levels in S-PHs plants (**Figure 3B**). Interestingly, several studies demonstrated that *TIP2;1* expression is reduced in grapevine leaves experiencing drought conditions (Pou et al., 2013; Dayer et al., 2020; Lukšić et al., 2023). These results correlate with water status measurements and indicate that S-H_2_O plants were more severely impacted by the stress conditions. The sustained expression of *TIP2;1* in PHs-treated plants suggests a possible contribution of PHs in the preservation of plant water transport capacity under combined heat and drought stress, thus facilitating rehydration of tissues upon stress relief. Interestingly, PHs application has been shown to induce the expression of several genes coding for aquaporins in *Citrus* plants exposed to salt stress (Lu et al., 2023).

These results demonstrate the potential of whey PHs to mitigate drought-induced physiological disruptions, possibly through mechanisms involving osmolytes accumulation and enhanced water uptake. Further research is needed to decipher the molecular pathways underlying PHs-mediated drought tolerance, particularly in field conditions. This will help determine the long-term benefits of PHs in enhancing grapevine resilience to drought stress and their broader implications for viticulture in the context of climate change.

### 4.2 Whey PHs impact pathogens development

Our results demonstrate that whey PHs impact *P. viticola* zoospore release and infectivity (**Figure 4**), while also affecting *B. cinerea* development *in vitro* and on grape berries when applied post-infection (**Figure 6**). These findings suggest that PHs exert a direct toxic effect on these pathogens. Despite this, PHs application triggered only a minor upregulation of defense-related genes, including *PR-1, PR-2, STS1*, and *PAL*, in grapevine leaves (**Figure 5**). This limited transcriptional response implies that whey PHs alone do not induce a substantial immune activation in grapevine after 24 h. However, it is possible that PHs initially triggered a stronger immune response, but gene expression returned to basal levels by the sampling timepoint. If the peak induction occurred earlier than measured, transient activation might have been missed. Future experiments with earlier timepoints post-treatment could clarify this. However, PHs-treated plants did not exhibit an enhanced defense response following pathogen challenge and compared to other conditions, suggesting that whey PHs do not function as priming agents under our experimental conditions. Furthermore, the lack of protective effects when PHs were applied prior to *B. cinerea* infection suggests that the immune-related effects of whey PHs are insufficient to confer strong resistance in grape berries. It is thus likely that the direct antifungal effects of whey PHs are the primary contributors to their efficacy.

Although the activation of *PAL* and *STS1* by PHs was minimal, these genes encode key enzymes in the biosynthesis of stilbenes like resveratrol, which are known to play a role in grapevine defense against *P. viticola* (Gabaston et al., 2017). Therefore, the slight activation of the phenylpropanoid pathway could still contribute to restricting *P. viticola* development, potentially through the accumulation of antimicrobial secondary metabolites. Future quantification of resveratrol and other stilbene compounds in response to whey PHs and pathogen infection could clarify their role in this process. Interestingly, our findings contrast with previous studies showing that casein- and soybean-derived PHs act as elicitors of grapevine immune responses and reduce *P. viticola* infection in leaves and *B. cinerea* in berries (Lachhab et al., 2014, Lachhab et al., 2016). These studies demonstrated upregulation of *PR-1*, *PR-2* and *STS* and induced stilbene accumulation in grapevine cells suspension. In contrast, our results suggest that whey PHs may act primarily as an oomycide/fungicide, with only a minor role as a resistance elicitor against *P. viticola*. This is in line with findings from a previous study (Krzyzaniak et al., 2018), although further research is needed to confirm this hypothesis.

The exact mechanism underlying the toxicity of whey PHs toward *P. viticola* and *B. cinerea* remains unknown. PHs produced via enzymatic hydrolysis typically contain amino acids and small bioactive peptides, which can exhibit antifungal activity (Malécange et al., 2023). While several studies have demonstrated the antifungal potential of PHs from various sources (Liu et al., 2007; Jeenkeawpieam et al., 2023; Alsaloom, 2024), their precise mode of action remains unclear. Research on peptide-pathogen interactions suggests potential mechanisms, including membrane permeabilization, disrupted sporulation, altered hyphal morphology, and direct interactions with fungal DNA (Muñoz and Marcos, 2006; Benfield and Henriques, 2020; Pirkhezranian et al., 2020). Given these findings, it is likely that the whey PHs used in our study contain bioactive peptides with similar effects on *P. viticola* and *B. cinerea*, however, their precise composition and mechanisms of action require further investigation.

## 5 Conclusion and future directions

This study demonstrates that whey-derived PHs enhance grapevine resilience to combined heat and drought stress by maintaining photosynthetic machinery, enhancing stress responses and accelerating recovery. Given the increasing incidence of heat waves and droughts in many winegrowing regions, whey PHs represent a promising biostimulant strategy to improve plant tolerance while reducing reliance on synthetic inputs. Additionally, their direct inhibitory effects on *P. viticola* and *B. cinerea* could contribute to lowering the use of synthetic and copper-based fungicides in viticulture, thereby promoting more sustainable disease management practices. Further studies should explore whether similar protective effects are observed with PHs derived from other protein sources, such as plant-based hydrolysates. Comparative analyses could provide insights into the specific bioactive peptides responsible for stress mitigation and determine whether certain PHs confer superior benefits under different environmental conditions.

The experiments were conducted under controlled climate chamber conditions, enabling precise regulation of environmental parameters and minimizing confounding factors. While these conditions ensured consistency and reproducibility across experimental trials, they differ from field environments in several key aspects. First, temperature and humidity remained stable throughout the stress period, whereas in vineyards, heatwaves and droughts vary in duration and intensity, with fluctuating environmental conditions potentially influencing plant responses and treatment efficacy. Additionally, potted plants were used, which may have influenced root development and water uptake compared to field-grown vines with deeper root systems. Despite these limitations, the controlled experimental approach was essential for dissecting specific physiological and molecular responses to whey PHs under combined heat and drought stress.

Likewise, while our controlled experiments demonstrated that PHs influence grapevine responses to *P. viticola* and *B. cinerea*, several challenges may arise when translating these findings to vineyard conditions. In our controlled conditions, pathogen inoculum levels, humidity, and temperature were carefully regulated to favor infection, allowing a precise evaluation of PHs’ effects on disease progression. However, disease outbreaks in vineyards are influenced by factors such as rainfall, temperature shifts and microbial competition. For instance, downy mildew development is highly dependent on prolonged leaf surface moisture, which can vary significantly across seasons and vineyard microclimates. Similarly, *B. cinerea* infections are strongly influenced by high humidity and wounding, both of which are less predictable in open-field conditions. Furthermore, vineyard conditions introduce additional stressors such as nutrient variability, mechanical damage from wind or hail, and simultaneous pressure from multiple pathogens. These factors may modify grapevine immune responses, potentially altering the effectiveness of PHs compared to controlled chamber conditions. To address these limitations, future research should include extensive field trials across diverse locations and multiple growing seasons to gain a deeper understanding of plant responses under variable conditions.

## Data Availability

The original contributions presented in the study are included in the article/**Supplementary Material**. Further inquiries can be directed to the corresponding author.
