# Supplementary material for: Whey protein hydrolysates enhance grapevine resilience to abiotic and biotic stresses
Source: Front Plant Sci. 2025 May 9;16:1521275. doi: 10.3389/fpls.2025.1521275 (PMC12098628; doi:10.3389/fpls.2025.1521275)
Supplement: Supplementary file 1 [file DataSheet1.docx]

Supplementary Figures


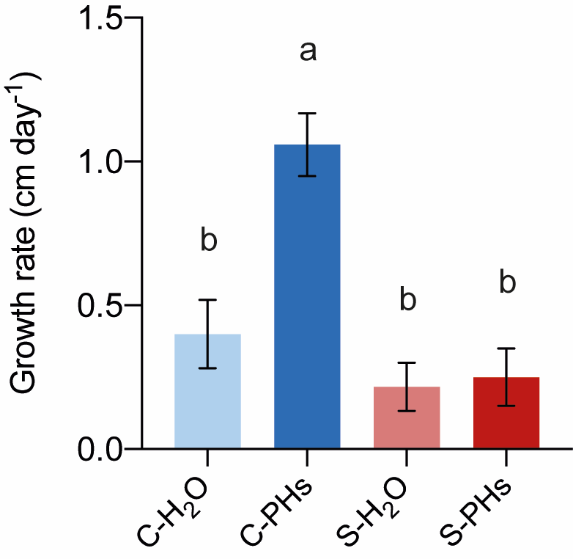


**Supplementary Figure 1.** Growth rate of *V. vinifera* plants under the different conditions. Data represent means ± SEM of three independent experiments (n=4 per experiment). Different letters indicate significant differences at *P* < 0.05 (One-Way ANOVA followed by Tukey’s Honest Significant Difference test).


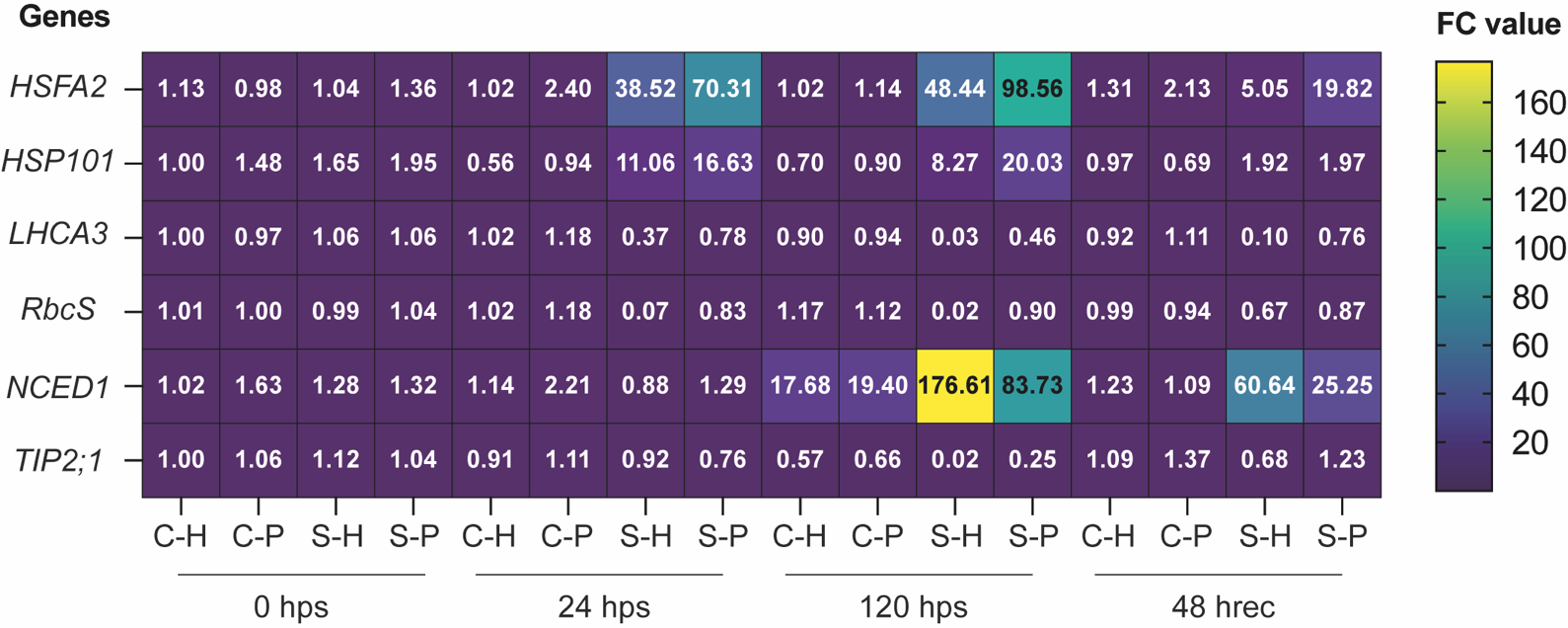


**Supplementary Figure 2.** Heat map of differential gene expression in response to PHs treatment and stress conditions. Values in cells indicate the fold change in gene expression across the different treatments and timepoints from Figure 2 and 3. Conditions are depicted according to the following abbreviations: C-H_2_O: C-H; C-PHs: C-P; S-H_2_O: S-H; S-PHs: S-P.

**
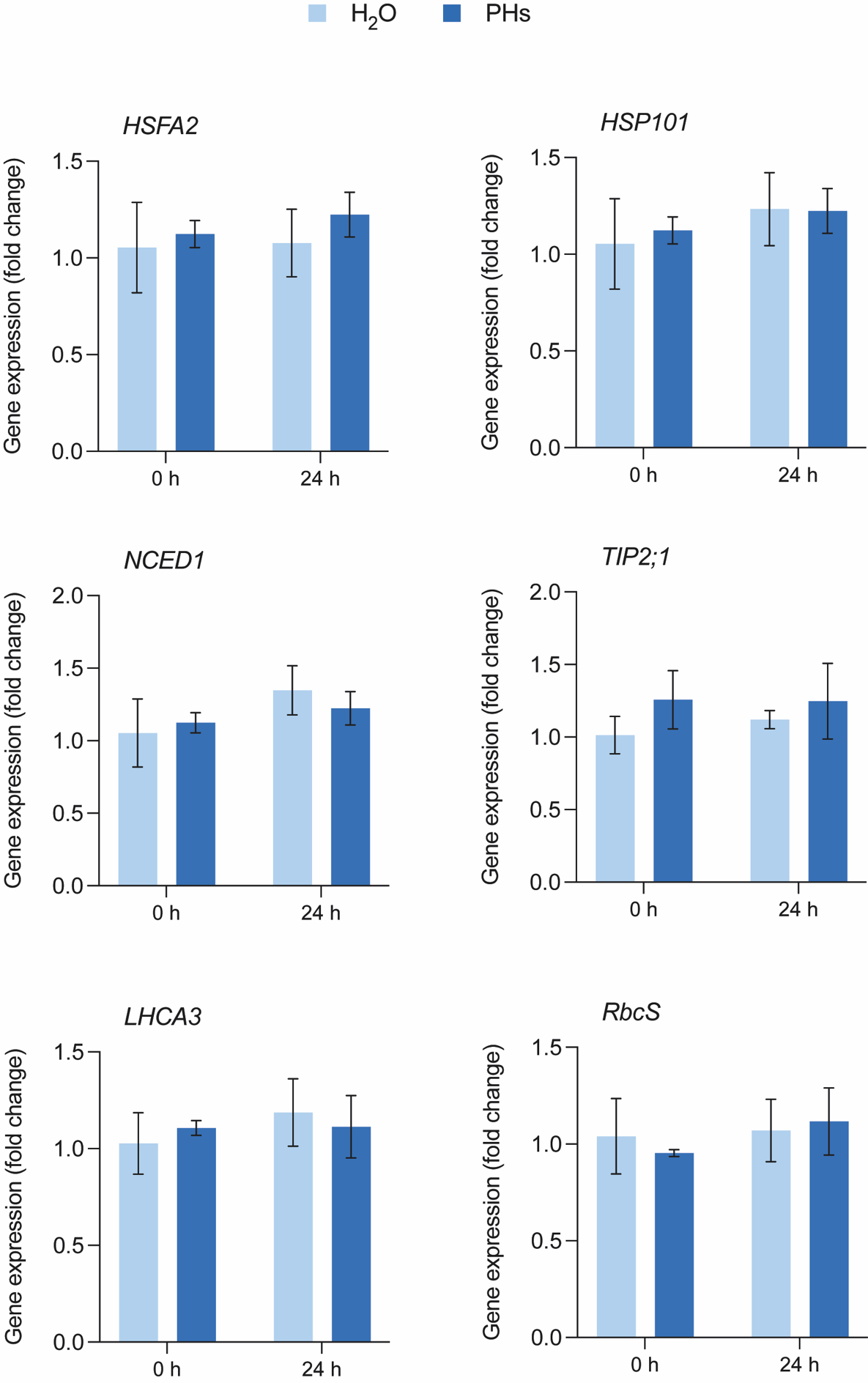
**

**Supplementary Figure 3.** Gene expression analysis in response to whey PHs application. Leaves were treated with water or whey PHs and gene expression levels were monitored 0 h and 24 h following treatment. Data represent means ± SEM of three independent experiments (n=3), each consisting of a pool of four leaves. No significant differences were observed.
